# Cooperative Regulation of Substrate Stiffness and Extracellular Matrix Proteins in Skin Wound Healing of Axolotls

**DOI:** 10.1155/2015/712546

**Published:** 2015-03-08

**Authors:** Ting-Yu Huang, Cheng-Han Wu, Mu-Hui Wang, Bo-Sung Chen, Ling-Ling Chiou, Hsuan-Shu Lee

**Affiliations:** ^1^Liver Disease Prevention and Treatment Research Foundation, Taipei 10002, Taiwan; ^2^Institute of Biotechnology, College of Bioresources and Agriculture, National Taiwan University, Taipei 10617, Taiwan; ^3^Department of Internal Medicine, National Taiwan University Hospital and National Taiwan University College of Medicine, Taipei 10002, Taiwan; ^4^Agricultural Biotechnology Research Center, Academia Sinica, Taipei 11529, Taiwan

## Abstract

Urodele amphibians (Ambystoma mexicanum), unique among vertebrates, can regenerate appendages and other body parts entirely and functionally through a scar-free healing process. The wound epithelium covering the amputated or damaged site forms early and is essential for initiating the subsequent regenerative steps. However, the molecular mechanism through which the wound reepithelializes during regeneration remains unclear. In this study, we developed an *in vitro* culture system that mimics an *in vivo* wound healing process; the biomechanical properties in the system were precisely defined and manipulated. Skin explants that were cultured on 2 to 50 kPa collagen-coated substrates rapidly reepithelialized within 10 to 15 h; however, in harder (1 GPa) and other extracellular matrices (tenascin-, fibronectin-, and laminin-coated environments), the wound epithelium moved slowly. Furthermore, the reepithelialization rate of skin explants from metamorphic axolotls cultured on a polystyrene plate (1 GPa) increased substantially. These findings afford new insights and can facilitate investigating wound epithelium formation during early regeneration using biochemical and mechanical techniques.

## 1. Introduction

Urodele amphibians (such as axolotls:* Ambystoma mexicanum*), unique among vertebrates, can regenerate appendages (tails and limbs) and other body parts (heart, spinal cord, lenses, and joints) entirely and functionally. The regeneration process in axolotls typically involves the following steps: (1) epithelial cells migrate from the cut edge and the wound epithelium forms, covering the amputated or damaged sites [1, 2]; (2) blastema is generated, containing undifferentiated and proliferating mesenchymal cells [3, 4]; and (3) in the later stage of regeneration, the blastema cells begin to redifferentiate and regrow in the original tissues and organs. The initiation of wound epithelium formation is crucial; however, various factors can hinder the regeneration process. For example, suturing a piece of skin on the wounded site immediately after amputation inhibits regrowth [5]. Unlike axolotls, mammals cannot regenerate most damaged organs and tissues, typically forming scars during the healing process instead; however, wound repair in fetal mammals and marsupials is scar-free [6–9]. Furthermore, the molecular and cellular developmental mechanisms of appendages and organs in vertebrates are similar and highly conserved to urodele amphibians. Therefore, it has been suggested that mammals can regenerate if particular factors or pathways activate downstream developmental signaling cascades [10]. Hence, comparing and investigating the differences in the early stages of wound healing between axolotls and mammals should enable insights into scar or scar-free wound healing in vertebrates and salamanders.

In axolotls and newts, the reepithelialization of wounded sites proceeds rapidly. Epidermal cells around the wound react and quickly migrate through pseudopodial projection [5, 11]. The expression of *β*1-integrin in the keratinocytes has been shown to support rapid migration on the wound bed [12]. Typically, the wounded site on juvenile axolotls can recover within 10 h [13] and even within 2 h on small axolotls [14, 15]. Unlike the rapid recovery in axolotls, the healing of a similarly sized wound in mammals takes 2 to 3 d [16, 17]. This delayed reepithelialization in vertebrates is similar to the early phase of wound healing in metamorphic axolotls that requires 72 h for the wounded area to be recovered [1]. In amphibians, the metamorphosis from a larva to a juvenile indicates the transformation of the physiological system. Axolotls are generally neotenic and do not undergo full metamorphosis into adulthood. Nonetheless, adding thyroxine in the rearing water [18] or administering a single shot through intraperitoneal (ip) injection induces metamorphosis [19]. The epidermal transformation from a pseudostratified to a stratified state following metamorphosis is assumed to impede the reepithelialization occurring in response to injury [1]. Moreover, increasing amounts of evidence have shown that changes in biomechanical environments determine cell fate and development [20–22]. In newts, the transition of extracellular matrices at various stages of limb regeneration leads muscle cells to proliferation, migration, fragmentation, and fusion. In addition, substrates with a softer and tenascin-C-coated environment increase the migration and fragmentation of the primary newt muscle cells; however, environments with a stiffer and laminin- and fibronectin-coated environment enhance cell differentiation [23].

In this research, we used an* in vitro *approach to investigate the early phase of an* in vivo *wound healing process in axolotls and the biomechanical properties in the system, including coated substances and degrees of substrate stiffness, both of which can be precisely defined and manipulated. Skin explants from the hind limbs of axolotls cultured on collagen-coated substrates ranging from 2 to 50 kPa rapidly reepithelialized within 10 to 15 h; however in harder (1 GPa) and other extracellular matrices (tenascin-, fibronectin-, and laminin-coated environments), the wound area recovered slowly. The reepithelialization rate of metamorphic axolotl skin explants cultured on a polystyrene plate (1 GPa) increased markedly. This advanced 2D culture system facilitates investigating the biochemical and mechanical mechanisms of wound epithelium formation during early regeneration in axolotls.

## 2. Materials and Methods

### 2.1. Animal Care

#### 2.1.1. Axolotl Rearing

Axolotls (*Ambystoma mexicanum*), kept in a continuous-flow aquaria system in separate cages, were subjected to 12 h light-12 h dark cycles; the temperature of the water ranged between 18°C and 20°C. The water was UV-treated and biofiltered to prevent contamination with microorganisms. The environment consisting of circulating water with a pH value of 7.7 to 8.0 and a conductivity of 500 to 750 *μ*S/cm was suitable for axolotl rearing. The axolotl larvae (<5 cm) were fed daily with brine shrimp (*Artemia*). The adult and juvenile axolotls (6–20 cm) were fed with fish pellets three times per week; leftovers were removed a few hours after feeding. The animals were anesthetized in a solution containing 0.1% MS222 (Sigma-Aldrich) before surgical procedures. Animal care and experimental procedures were approved by the Institutional Animal Care and Use Committee of the National Taiwan University College of Medicine.

#### 2.1.2. Axolotl Metamorphosis

Axolotls are generally neotenic and reach adulthood without undergoing metamorphosis. However, we were able to induce metamorphosis using thyroxine (T_4_), which was added to their rearing water [18] or administered through ip injection [19]. We intraperitoneally injected 1.5 *μ*g of T_4_/g (axolotl body weight) in juvenile axolotls (10–12 cm). Within 10 to 12 d, the axolotls underwent complete metamorphosis.

### 2.2. *In Vitro* Skin Culture

#### 2.2.1. Skin Explants Preparation

The skin explants were prepared in a culture hood. After the hind limbs of the axolotls were amputated, the full-thickness skin (including dermis and epidermis) covering the hind limbs was carefully removed with fine forceps and scissors and rinsed as follows: the skin was rinsed with 70% ethanol for 10 s and then with amphibian phosphate buffered saline (APBS: 60% 1x PBS) three times for 30 s per rinse. The skins were cut into 4 × 4 mm pieces in an APBS buffer, and a 2 mm hole was created in the center of the skin explants using a sterile biopsy punch (Miltex). Subsequently, the explants were transferred to 12 well culture plates featuring various biomechanical environments, and a 100 *μ*L culture medium (60% Dulbecco's Modified Eagle Medium; 10% fetal bovine serum; 1x Pen/Strep and ITS; 10 *μ*g/mL of insulin, bovine pancreas; 5.5 *μ*g/mL of human transferring; and 5 ng/mL of sodium selenite; Sigma-Aldrich) was applied to the center of the hollowed explants to retain moisture. After incubation at 22°C in an atmosphere with 1% CO_2_ and 99% air for 2 to 3 h, 200 *μ*L of additional culture medium was added to the wells. The samples were then ready for further observation using a real-time recording microscopy system.

#### 2.2.2. Generation of Biomechanical Environments

We used hydrogels with four levels of stiffness (0.2, 2, 12, and 50 kPa, Matrigen, Brea, CA). The wells were coated with different substrates, such as tenascin (2 *μ*g/cm^2^; Millipore CC065), fibronectin (2 *μ*g/cm^2^; Millipore FC010), laminin (2 *μ*g/cm^2^; Millipore CC095), and collagen type I rat tail (20 *μ*g/cm^2^; 354236, BD Biosciences, Bedford, MA, USA), and diluted in PBS (1x) at 37°C. After ≥1 h of incubation, the wells were gently rinsed three times with PBS (1x) to remove excess substrates and stored at 4°C. Before the skin explants were transferred, the wells were rinsed with a culture medium three times for 30 min to rebalance the substrate. Two concentrations of collagen type I (20 *μ*g/cm^2^ and 300 *μ*g/cm^2^) were coated onto the polystyrene plate (24 wells, 3524, Corning Life Science) that exhibited a 1 GPa stiffness.

### 2.3. Time-Lapse Microscopy for* In Vitro* Analysis

To monitor and record the entire process of wound closure in the skin explants, time-lapse microscopy was used. The plates with cultured skin explants were placed on a Laser TIRF 3 (Carl Zeiss) with high-resolution CCD (AxioCam MRm 1388∗1040P), and images were taken at 5x magnification every 15 min for 15 h in* x*-,* y*-, and* z-* axes. Images were analyzed using AxioVision software and exported as AVI or TIFF files. The reepithelialization area was evaluated periodically using ImageJ software.

## 3. Results 

### 3.1. Analysis of Skin Explants in Biomechanical Environments

#### 3.1.1. Skin Explants on Soft Tissue-Like Substrate

**Figure 1:**
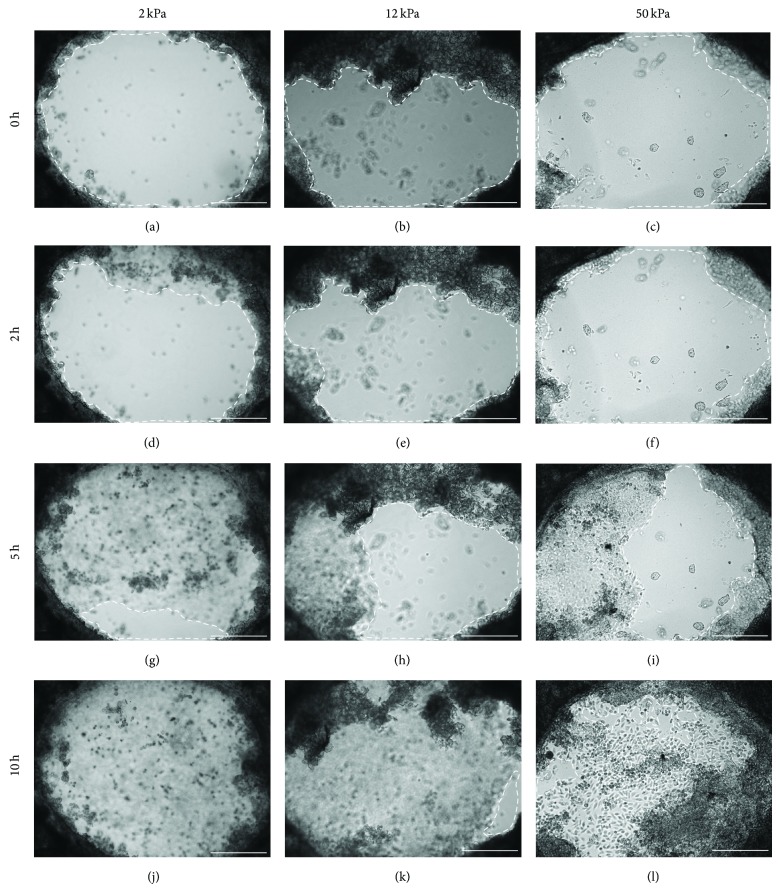
*In vitro* culture of axolotl skin explants on substrates with various degrees of stiffness. All skin explants initially exhibited 2 mm punched holes; images of reepithelialization were recorded every 15 min. The unrecovered area is circled by a dashed line. Three degrees of substrate stiffness (2, 12, and 50 kPa) were investigated, respectively, from (a) to (c), (d) to (f), (g) to (i), and (j) to (l). From 0 to 2 h, most of the area was still uncovered in (a) to (c) and (d) to (f). After 2 h, the reepithelialization rate increased and more area was covered in (g) to (i); healing was complete within 10 h in (j) to (l); scale bars: 500 *μ*m.

**Figure 2:**
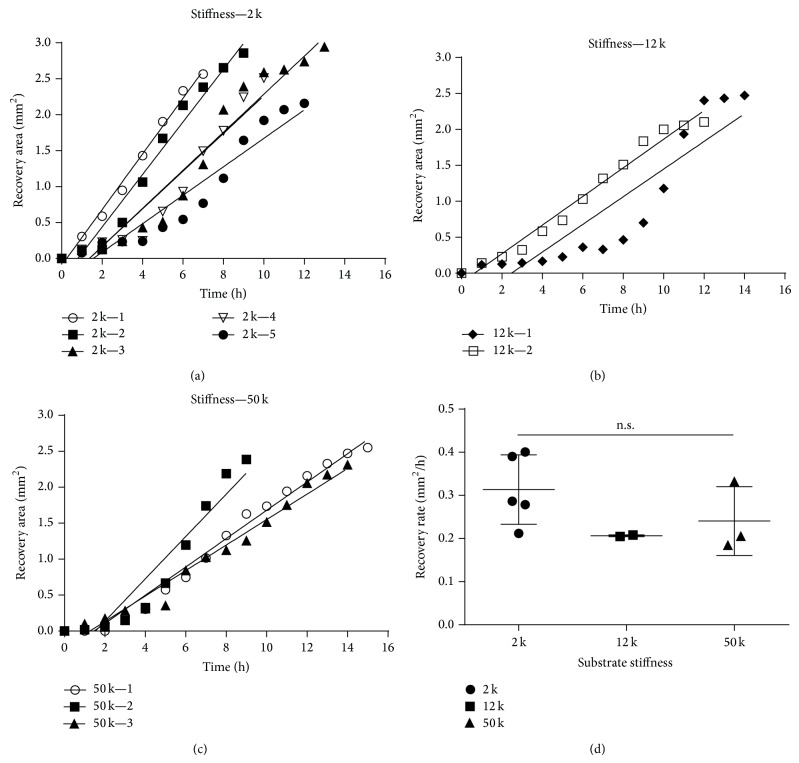
Quantitative analysis of the skin-wound closure rate in substrates with various degrees of stiffness. Sequential images of the complete reepithelialization of skin explants were recorded for 15 h. Levels of skin recovery are shown for three degrees of substrate stiffness: five of six, two of six, and three of six skin explants in (a), (b), and (c), respectively, completely healed within 15 h. The keratinocytes migrated little during 0–2 h, and the average wound closure rate was between 0.18 and 0.4 mm^2^/h. The average migration rate was not significantly different between these treatments by unpaired *t*-test (d).

Recent studies have indicated that cell development or differentiation responds to changes in the stiffness of the microenvironment [22, 24–26]. To investigate the mechanism regulating rapid wound closure in axolotls, we recorded the reepithelialization process of skin explants cultured on various collagen-coated substrates with varying degrees of stiffness ranging from 2 to 50 kPa (Figure 1), which were similar to the stiffness of various organs, such as skeletal muscles, arteries, and skin [22]. The keratinocytes near the wound bed migrated little during a period of 1-2 h (Figures 1(a)–1(f); Figures 2(a)–2(c)). The average migration rate of keratinocytes ranged between 0.18 and 0.4 mm^2^/h, and there was no significant difference of the average migration rates of these skin explants cultured on various degrees of substrate stiffness with collagen-coated wells (Figure 2(d)). Once most of the keratinocytes began to migrate, they typically did so at a consistent migration rate. However, we noticed an abruptly increase in the migration rate in few skin explants during the period of 6–10 h (Figure 2(a), filled circle; Figure 2(b), filled diamond). The wound-closure time in the cultured skins typically lasted 12 h and was comparable to the wound healing* in vivo* with a 1.5 mm punch [2] and a 4 mm punch [1], and the wound healing in an* ex vivo* culture with a 2 mm punch [27]; the recovery time of each wound lasted approximately 8, 24, and 11 h, respectively. Increasing the size of the wound area twofold approximately doubled the healing time.

#### 3.1.2. Skin Explants on Hard Tissue-Like Substrate

**Figure 3:**
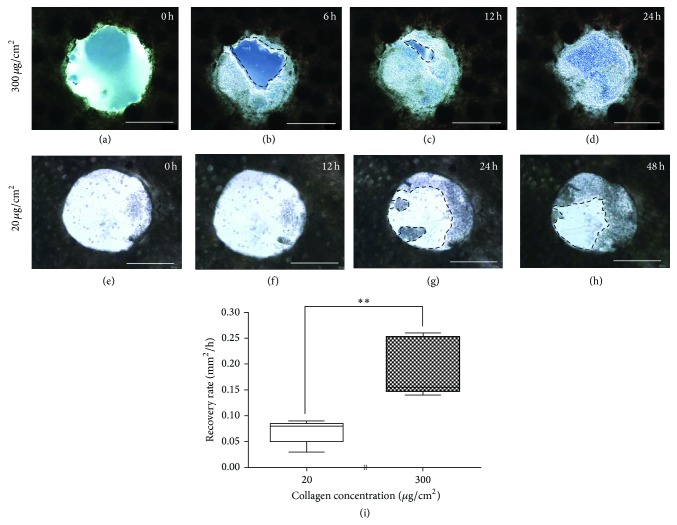
*In vitro* culture of axolotl skin explants on a polystyrene plate. Skin explants with 2 mm punch holes were cultured on 300 *μ*g/cm^2^, (a) to (d), and 20 *μ*g/cm^2^, (e) to (h) collagen-coated petri dishes. Images of the wound closure were obtained within 24 h (a) to (d) and 48 h (e) to (h). After culturing for 48 h, the wounded areas remained unable to recover completely on 20 *μ*g/cm^2^ collagen-coated polystyrene plate (h, dashed line). Cultured wound epithelium showed significant difference of average migration rate after 6 h incubation by unpaired *t*-test (i). Sample sizes for each condition were as follows: 20 *μ*g/cm^2^ for 6 h culture: *N* = 5; 300 *μ*g/cm^2^ for 6 h culture: *N* = 5. ^**^ *P* = 0.0018. Scale bars: 1000 *μ*m.

With the substrate stiffness from 2–50 kPa, the average migration rate of skin explants showed no significant difference (Figure 2(d)). To test the ability of the skin to recover in a harder environment, skin explants were cultured directly on a 20 and 300 *μ*g/cm^2^ collagen-coated polystyrene plate. The former substrate featuring a stiffness of 1 GPa, which was approximately six orders of magnitude stiffer than the latter with stiffness of 1 kPa [28]. In a hard environment, keratinocytes migrated little in the initial 12 h (Figures 3(e) and 3(f)), whereas they were almost covered around the wounded area in a culture exhibiting a softer environment (Figure 3(c)). Between 12 and 24 h, the keratinocytes began to migrate slowly from the wound edge (Figures 3(f) and 3(g)) and already recovered completely in soft environment (Figure 3(d)). Comparing the average migration rate of the wound epithelium cultured on hard and soft environments showed significant difference after 6 h incubation (Figure 3(i)).

#### 3.1.3. Skin Explants in Extracellular Matrices-Coated Environments

To test whether the degree of stiffness and extracellular matrix proteins combined integrated signals to involve in the migration of epithelial cells, the three additional extracellular matrix proteins, tenascin, fibronectin, and laminin, were incorporated on soft substrate to culture skin explants. On substrate coated with tenascin and laminin, the skin explants scarcely covered the wounded area, and only partial reepithelialization occurred on the fibronectin-coated substrate (Table 1). Regarding the substrate coated with collagen, the softest substrate (0.2 kPa stiffness) exhibited only partial recovery. Skin explants cultured on substrate featuring 2 kPa stiffness exhibited the highest degree of full recovery (83.3%; *n* = 5 in 6) compared with other substrates featuring stiffness of 12 kPa (33.3%; *n* = 2 in 6) and 50 kPa (50%; *n* = 3 in 6) (Table 1).

### 3.2. Analysis of Skin Explants from Metamorphic Axolotls

**Figure 4:**
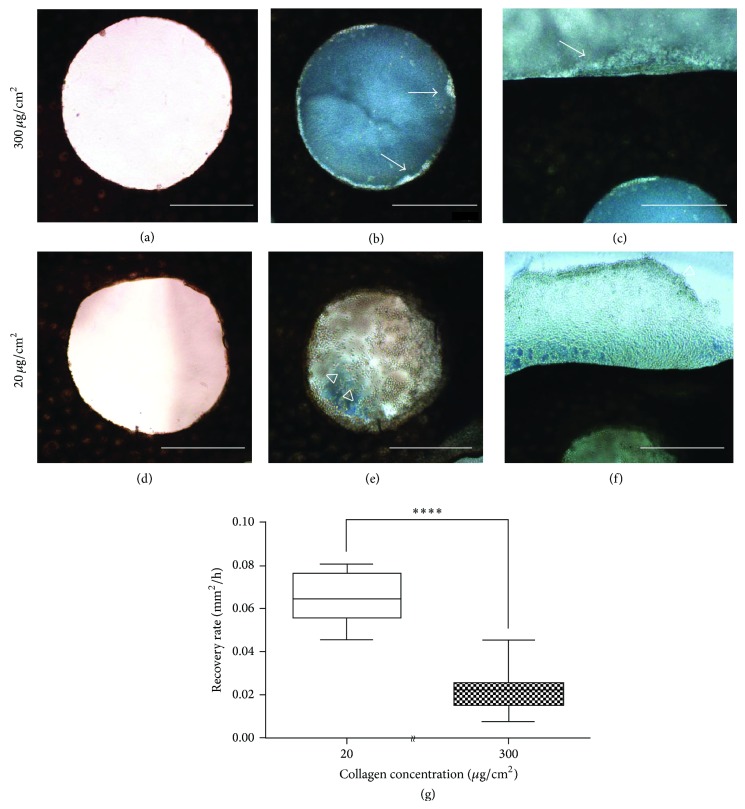
*In vitro* culture of metamorphic axolotl skin explants on polystyrene plate. Skin explants with 2 mm holes were cultured on 300 *μ*g/cm^2^, (a) to (c), and 20 *μ*g/cm^2^, (d) to (f), collagen-coated plate. After 24 h of culturing, the keratinocytes migrated little, neither from the inner punched holes (b, arrows) nor from the outer edge of the skin explants (c, arrow) on the 300 *μ*g/cm^2^ collagen-coated petri dish. By contrast, on the 20 *μ*g/cm^2^ collagen-coated petri dish, the keratinocytes migrated further, both inner and outer from the skin explants (e and f, hollow arrow heads). Wound epithelium recovery showed significant difference of average migration rate after 24 h incubation by unpaired *t*-test (g). Sample sizes for each condition were as follows: 20 *μ*g/cm^2^: *N* = 6; 300 *μ*g/cm^2^: *N* = 9. ^****^ *P* < 0.0001. Scale bars: 1000 *μ*m.

The reepithelialization in the initial phase of wound healing in metamorphic axolotls was complete within 72 h, and the compositions and microenvironments near the epithelium presumably changed because of the metamorphosis [1]. To investigate whether the collagen-coated and substrate stiffness regulated the wound-epithelium recovery in metamorphic axolotls, we cultured skin explants on a polystyrene plate coated with two concentrations of collagen: 20 (1 GPa stiffness) and 300 (1 kPa stiffness) *μ*g/cm^2^. The skin explants cultured for 24 h with 300 *μ*g/cm^2^ of collagen exhibited minimal wound epithelium migration near the wound edge (Figures 4(b) and 4(c), arrows). By contrast, in the skin explants cultured with 20 *μ*g/cm^2^ of collagen, the epithelium recovered most of the wound area (Figure 4(e), hollow arrowheads). The epithelial cells had moved inside, filling the wound area, and also progressed to the outside of the skin explants (Figure 4(f), hollow arrowheads). In addition, the average migration rate of the metamorphic wound epithelium was shown to be significantly different on hard and soft environments after 24 h culture (Figure 4(g)).

## 4. Discussion and Conclusion

In mammals, wound healing is a natural self-defense process that prevents microorganisms from intruding an open wound and leads to the formation of a collagenous scar at the wounded site [29]. Although wound closure is essential for maintaining tissue integrity, scars impair movement and can impede the function of some tissues and organs [30]. Unlike mammals, axolotls are capable of scar-free healing. The rapid onset and activation of keratinocyte migration within the first few hours of the initial phase of wound healing have been suggested to cause rapid reepithelialization in axolotls that is typically fivefold to tenfold faster than that in vertebrates [16, 17, 31]. However, it is a challenging task to track the whole wound healing process in adult axolotls, and there was no suitable cell line for further investigation. Developing an* in vitro* culture system was able to bridge the gap between the animal experiment and cell culture approach. In this study, we cultured skin explants from axolotl hind limbs, controlling parameters, including substrate stiffness and extracellular matrix proteins. According to our results, the wounded skins with a 2 mm punch recovered in 12 h with reepithelialization rates of 0.18 to 0.4 mm^2^/h. These results are comparable to those of two recent* in vivo* studies: recovery of skin with a 1.5 mm punch required <8 h [2]; recovery of skin with a 4 mm punch required <24 h [1]. In addition, in an* in vitro* study, recovery of skin with a 2 mm punch required <11 h [27], suggesting that the* in vitro* approach simulated the wound healing process in axolotls. Most of the skin explants exhibited a constant rate of healing in the wound area (Figure 2); few explants exhibited accelerating reepithelialization during the wound recovery process (Figure 2(a), filled circle; Figure 2(b), filled diamond). This abrupt change in the migration rate suggests that there is an interaction between the collective epithelial cells and the surrounding microenvironment. The adherent collective cells have been shown to exert a strong traction force on the anchor site of the matrix [32–34] and surrounding cells [35–38]; the stress response from the matrix might alter the cytoskeleton arrangement and network in the cell. This synergistic cooperation between the wound-healing epithelial cells and their environment is believed to play a role in the rapid recovery of the wound area in axolotls. Future experiments will be needed to identify the genes during various healing stages and determine molecules that might stimulate or activate the onset of keratinocyte migration in the early phase of axolotl wound healing.

In the softest environment in our experiments, none of the skin explants (*n* = 6) with a substrate stiffness of 0.2 kPa and collagen coat recovered completely; however, most of the wounded skins healed completely when substrate stiffness exceeded 2 kPa (Table 1). We did not observe the recovery of the wounded skin cultured in a tenascin- (*n* = 6) or laminin-coated (*n* = 6) environment with various degrees of substrate stiffness and noted only a partial recovery in the fibronectin- (*n* = 2 in 6, 33%) coated environment (Table 1). However, the* in vitro* culture of the primary newt myoblasts exhibited a higher migration rate and a preferential muscle fragmentation in the tenascin-rich environment with a substrate stiffness of 2 and 15 kPa [23]. In vertebrates, mammalian epithelial cells exhibit an increase in migration speed with increasing stiffness [25]. To investigate skin recovery in a harder environment, we cultured wounded skins on a polystyrene collagen-coated plate featuring a 1 GPa stiffness. After 48 h of culturing, recovery was not fully completed, suggesting that a harder material or environment impedes epithelial cell migration in axolotls (Figure 3). The sensitivity of cells to biophysical cues was also observed in early stage of mouse embryo cultures [24]. The percent development, hatching frequency, and cell number in the mouse-embryo development were affected when culturing was performed with a substrate stiffness of 1 kPa and 1 GPa. In sum, in different species and cell types, substrate stiffness and environments where cells contact with might affect the physical or morphology behavior of the cells. This suggests that creating a microenvironment that mimics* in vivo* conditions is feasible when appropriate combinations of substrate stiffness and extracellular matrices are used.

Adult mammals are incapable of healing in full-thickness skin wound damage, whereas fetal mammals can recover from similar types of wounds through scar-free healing [7]. This phenomenon has also been observed in amphibians and anurans undergoing metamorphosis heal wounds through scar formation [39]. However, tadpoles and pre- and postmetamorphic urodeles exhibit limitations such as embryonic development [40–42], body size [43–46], and aging [47, 48] for further wound healing investigation. Compared with tadpoles and urodeles, axolotls feature an ideal system for investigating wound healing because metamorphosis can be induced experimentally, and the age-associated factors can be controlled. In this paper, we describe the recovery of full-thickness skin wounds on axolotl limbs using an* in vitro *culture approach in paedomorphs (Figure 1) and in metamorphs (Figure 4); the wounded skin from metamorphic axolotls exhibited delayed reepithelialization within 24 h. This is similar to the* in vivo *wound healing of metamorphic axolotls observed by Seifert et al. in 2012 [1]. In addition, we cultured the metamorphic wounded skin in a hard environment and discovered that the reepithelialization rate substantially increased (Figure 4(g)). We hypothesized that the change in epigenetic regulation might be determined by the axolotl metamorphosis and result in various healing processes, affecting scar formation and the specific reaction to the microenvironment of cells. In mammals, the epigenetic regulation exerts a strong influence on the phenotype of myofibroblast, which is involved in fibrogenesis and scar formation [49]. Furthermore, the interaction between DNA methylation and MeCP2 is crucial for the differentiation and fibrosis of myofibroblast in the liver and lungs [50, 51]. Therefore, comparing the epigenetic profiling during the healing process in paedomorphic and metamorphic axolotls might yield insights into the rapid reepithelialization and scar-free wound healing of axolotls. Furthermore, investigating controllable factors such as substrate stiffness and extracellular matrix proteins shown in this study was able to alter reactions of cells in* in vivo* environment.

Our findings reveal that the reepithelialization during the wound healing process in axolotls can be mimicked by using an* in vitro *approach and the cell behavior are regulated by controlled extracellular matrix proteins and substrate stiffness. These findings indicate that integrated cues from the microenvironment affect the cell reaction, for example, accelerating or impeding the cell migration. Furthermore, using this approach, we not only successfully repeated the* in vitro* wound healing process developed by Ferris et al. (2010) [27] in our system but also demonstrated the mechanical and biochemical properties regulating the plasticity of epithelial cells.

## Figures and Tables

**Table 1:** Effect of substrate stiffness and extracellular matrix proteins on wound closure in skin explants culture.

	Stiffness (kPa)	Number of skin explants	Complete wound closure	Partial wound closure	No wound closure
Collagen (20 *μ*g/cm^2^)	0.2	6	0	4	2
	2	6	5	1	0
	12	6	1	5	0
	50	6	2	4	0

Tenascin (2 *μ*g/cm^2^)	0.2	6	0	0	6
	2	6	0	0	6
	12	6	0	0	6
	50	6	0	0	6

Fibronectin (2 *μ*g/cm^2^)	0.2	6	0	0	6
	2	6	0	0	6
	12	6	0	0	6
	50	6	0	2	4

Laminin (2 *μ*g/cm^2^)	0.2	6	0	0	6
	2	6	0	0	6
	12	6	0	0	6
	50	6	0	0	6

^*^Skin explants were sliced into 4 × 4 mm^2^ and punched 2 mm hole in the center.

^*^Wound closure was determined and recorded after 15 h culture.
